# Association of left atrial strain and fibrin clot properties in patients with heart failure and severe mitral regurgitation undergoing transcatheter edge-to-edge repair

**DOI:** 10.1007/s11239-025-03169-0

**Published:** 2025-08-29

**Authors:** Aleksandra Woźniak, Andrzej Gackowski, Karolina Golińska-Grzybała, Barbara Szlósarczyk, Jarosław Trębacz, Jadwiga Nessler, Grzegorz Gajos, Aleksander Siniarski

**Affiliations:** 1 Department of Coronary Artery Disease and Heart Failure, St. John Paul II Hospital, Krakow, Poland; 2 Noninvasive Cardiovascular Laboratory, St. John Paul II Hospital, Krakow, Poland; 3 Department of Coronary Artery Disease and Heart Failure, Institute of Cardiology, Faculty of Medicine, Jagiellonian University Medical College, Krakow, Poland; 4 Department of Interventional Cardiology, Institute of Cardiology, Faculty of Medicine, Jagiellonian University Medical College, Krakow, Poland

**Keywords:** Fibrin clot, Heart failure, Left atrial strain, Mitral regurgitation, Thrombin generation

## Abstract

The link between heart failure (HF) and increased prothrombotic risk has gathered attention, with several studies exploring this association. Patients with HF and severe mitral regurgitation (MR) undergoing transcatheter edge-to-edge repair (TEER) may present enhanced left atrial (LA) function and improve thrombosis-related factors due to the procedure. This study investigates the role of left atrial strain (LAS), assessed via speckle-tracking echocardiography, in detecting subtle LA abnormalities and its potential link to thrombotic risk in severe MR patients. 31 consecutive patients with severe MR who underwent TEER were enrolled. Six patients were lost to follow-up, and 25 completed both visits (V1 and V2). Coagulation parameters (thrombin generation, clot permeation [Ks], clot lysis time [CLT]) and echocardiographic assessments were performed at each visit. Subgroup analysis was also done for patients with sinus rhythm and atrial fibrillation. A significant correlation was found between LAS-r and changes in CLT (*p* = 0.03; *R* = -0.43) before and after TEER. LAS-cd decreased, and LAS-ct increased. No significant differences were noted in coagulation parameters. Patients with sinus rhythm also showed a significant correlation with CLT changes. This study demonstrates significant changes in LAS after TEER, with reduced LAS-cd and increased LAS-ct, which highlights a potential link between cardiac mechanics and clotting properties, with distinct differences between patients with sinus rhythm and atrial fibrillation. These findings provide insights into cardiac and thrombotic changes post-TEER, requiring further investigation.

**Graphical abstract:**

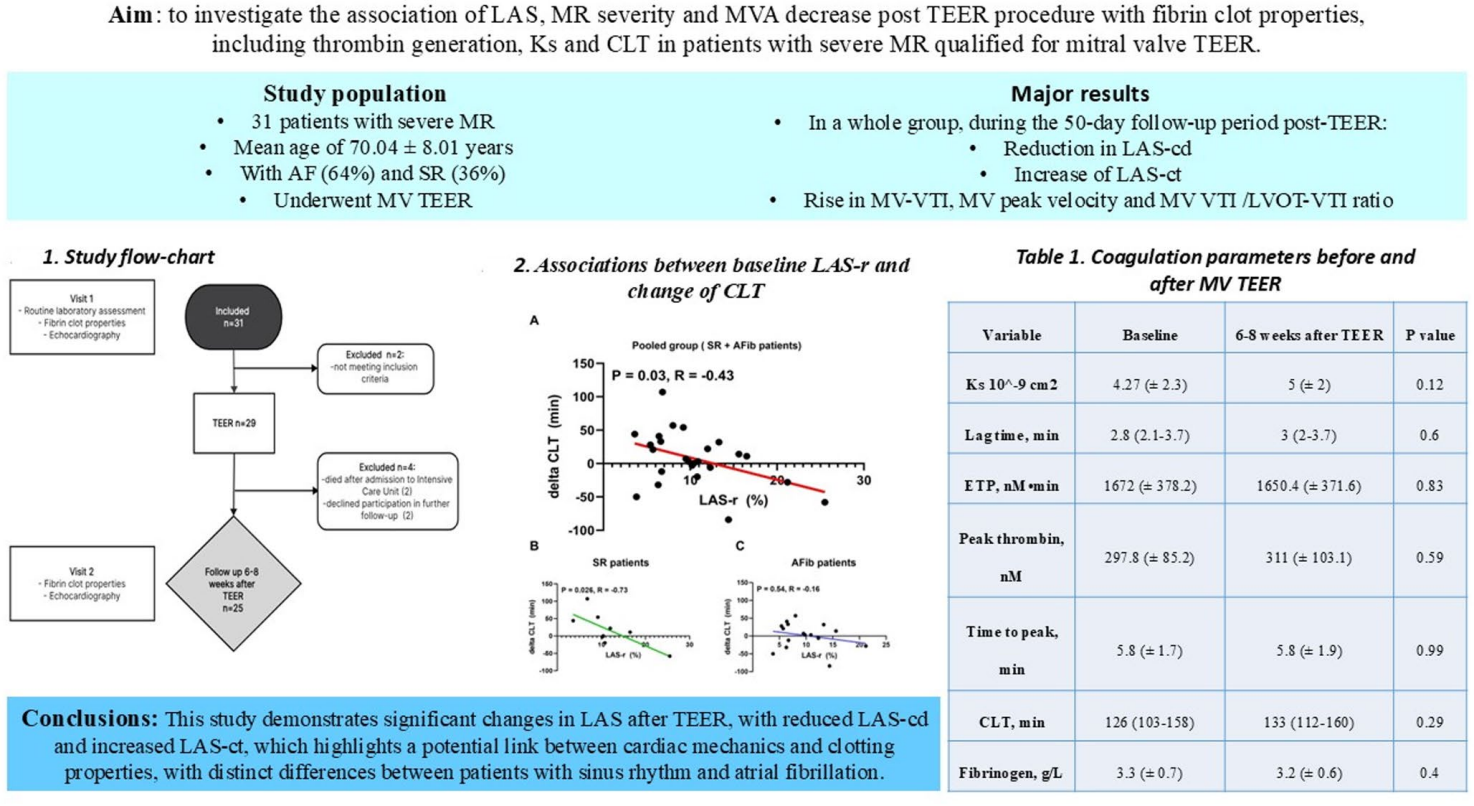

**Supplementary Information:**

The online version contains supplementary material available at 10.1007/s11239-025-03169-0.

## Highlights

- Left atrial strain (LAS) dynamics were assessed via speckle-tracking echocardiography in severe mitral regurgitation (MR) patients undergoing TEER.
- LAS changes after TEER: LAS-cd decreased, while LAS-ct increased, reflecting altered atrial mechanics.
- Thrombotic risk link: A significant correlation was observed between LAS-r and clot lysis time (CLT) (p = 0.03; R = –0.43).
- Rhythm-specific findings: Patients in sinus rhythm showed stronger associations between LAS parameters and CLT changes compared to those with atrial fibrillation.
- Coagulation properties: thrombin generation, clot permeation, and CLT showed no overall significant group-level changes.
- Potential clinical implication: LAS assessment may provide insights into the interplay between atrial mechanics and thrombotic risk after TEER.

## Introduction

The association between heart failure (HF), especially in acute HF settings and an increased prothrombotic risk has been investigated and previously described [1, 2]. Robust evidence has been accumulated, demonstrating that patients with HF have increased risk of thrombotic events due to the accelerated generation of densely packed fibrin clots [2–7]. Whereas the impact of mitral regurgitation (MR) on thrombin generation and fibrin clot properties remain poorly studied. Research show that while increased atrial flow may reduce clot formation and D-dimer levels, turbulent jets can promote coagulation via shear-induced activation [8, 9]. Patients with severe MR undergoing transcatheter edge-to-edge repair (TEER) represent an interesting observational group due to the minimally-invasive nature of the TEER procedure and the significant impact of reducing the severity of MR on left atrial (LA) function and LA blood hydrodynamics, which may potentially lead to changes in thrombosis-related parameters.

Left atrial strain (LAS) utilizes the speckle-tracking echocardiographic technique to show the alteration in the LA geometric configuration and deformation throughout the cardiac cycle. This approach enables the evaluation of subtle functional anomalies in the LA wall such as impaired relaxation or stiffness [10]. Furthermore, the role of LAS in the context of MR remains insufficiently determined while initial investigations suggested its promise as a metric for a comprehensive evaluation of cardiovascular (CV) outcomes in individuals with severe MR [11, 12].

There is currently no direct evidence specifically linking LAS parameters with detailed coagulation abnormalities. However, an increasing literature shed the light on the association between LAS and an elevated thrombotic risk, including an increased likelihood of stroke events or mortality following cryptogenic stroke, as well as the prediction of postoperative stroke [13–17]. Broader research does show an association between LA dysfunction and a prothrombotic state, though mostly in the context of atrial fibrillation [18, 19]. Assessment of LA function is therefore crucial to properly understand thromboembolic risk in this patient cohort. On the other hand, the decreased LA flow velocities resulting from elimination of severe mitral regurgitation and lowering of the mitral valve orifice area after TEER procedure may also affect the clotting properties.

This prospective study aimed to investigate the association of LAS, MR severity and mitral valve area decrease post TEER procedure with fibrin clot properties, including thrombin generation, clot permeation and clot lysis time (CLT) in patients with severe MR qualified for mitral valve TEER. We hypothesize that the TEER procedure, by reducing the MR, will affect the fibrin clot properties and thrombin generation.

Given the differences in left atrial function between patients with atrial fibrillation and those in sinus rhythm, the study will present the data both in aggregate and stratified by rhythm status to ensure accurate interpretation and subgroup-specific analysis.

## Methods

### Study design

**Fig. 1:**
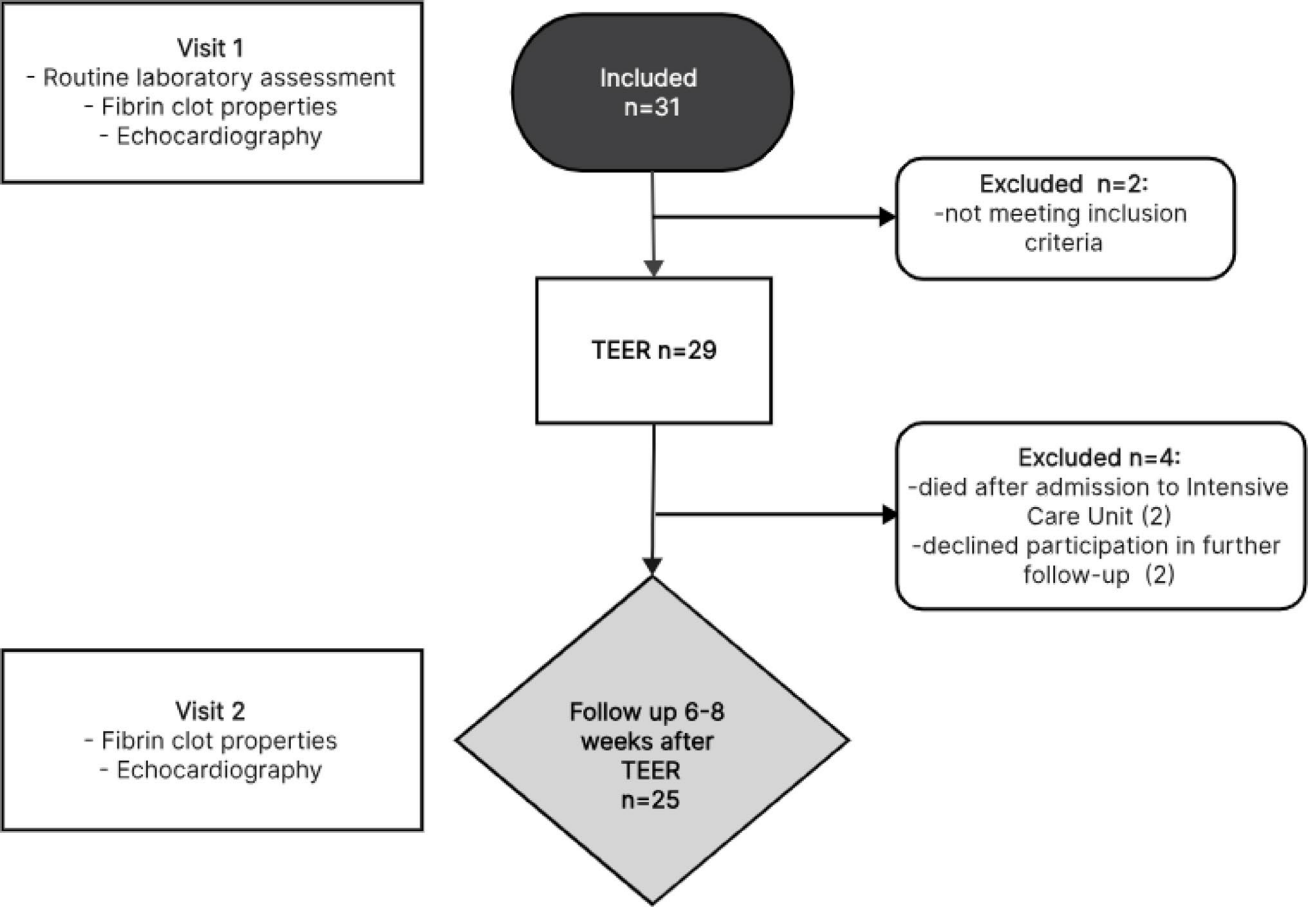
Research flow-chart. TEER; transcatheter edge-to-edge repair

This was a prospective study conducted in the Department of Coronary Artery Disease and Heart Failure, and in dept of Interventional Cardiology, Institute of Cardiology, Faculty of Medicine, Jagiellonian University Medical College. The present study was designed to follow the guidelines outlined in the Declaration of Helsinki, and its protocol received approval from the Jagiellonian University Bioethical Committee (No.: 1072.6120.10.2022 dated 26 Jan 2022). All enrolled patients underwent echocardiographic evaluation and comprehensive assessment of laboratory and coagulation parameters before and after TEER. Post-procedural visit was predefined as part of a standardized follow-up protocol. Flowchart of the study is demonstrated in Fig. 1.

### Patients

We initially included 31 patients with severe MR and symptomatic HF who were admitted to the St. John Paul II Hospital in Kraków, Poland between March 2022 and April 2023 to undergo the mitral valve TEER procedure. Detailed inclusion and exclusion criteria are available in Table 1.

**Table 1:** Detailed inclusion and exclusion criteria

Inclusion criteria	Exclusion criteria
age ≥ 18 years symptomatic, established diagnosis of HF severe mitral regurgitation feasibility for the TEER procedure a positive Heart Team qualification for the procedure.	vitamin K antagonists treatment heparin or bivalirudin administration less than 24 h prior to blood sampling active bleeding known coagulation system pathologies platelet count ≤ 100 × 10^9 diagnosed significant liver dysfunction (1.5 times above upper reference limit of alanine or aspartate transaminase) renal failure (estimated glomerular filtration rate [eGFR] < 30 ml/min/1.72 m^2) chronic treatment with non-steroidal anti-inflammatory drugs (excluding low-dose aspirin) systemic steroid therapy

MR, mitral regurgitation; TEER, Transcatheter Edge-to-Edge Repair

### Echocardiography

The transthoracic echocardiographic examination was conducted using the Philips Epiq 7 device (Philips Medical Systems, Bothell, WA) equipped with a sector probe (5 MHz). Measurements recorded during the examination were performed in accordance with the latest guidelines in six classical projections: parasternal long axis (PLAX), parasternal short axis (PSAX), apical 4-chamber (Ap4Ch), apical 5-chamber (Ap5Ch), apical 2-chamber (Ap2Ch), and apical 3-chamber (Ap3Ch).

The measurement of left atrial strain (LAS) was performed according to European Association of Cardiovascular Imaging (EACVI) in the Ap4Ch view utilizing speckle-tracking technique and calculated by AutoStrain LA - a specialized software package, with manual adjustments if required [20]. The analysis utilized the QRS complex as a zero-reference point, enabling assessment of LAS in 3 phases: LAS in reservoir phase (LAS-r) during early atrial diastole; LAS conduit phase (LAS-cd) during atrial mid-diastolic, and LAS contraction phase (LAS-ct) during atrial systole. Routine echocardiographic assessment, included: left atrial area (LA area), indexed left atrial volume (LAVI), left ventricular ejection fraction (LVEF), left ventricular end-diastolic (LVEDV) and end-systolic volumes (LVESV), left ventricular global longitudinal strain and the right ventricle assessment shown in Table 2 in detail, were determined in the Ap2Ch, Ap4Ch and Ap3Ch views. The LAS assessment was performed by two independent echocardiography experts. Mitral regurgitation (MR) was qualified as primary or secondary. MR severity was assessed according to American Society of Echocardiography, grading the MR severity as none/trace, mild, moderate or severe, both prior to and after TEER procedure.

**Table 2:** Baseline characteristics, laboratory evaluation and pharmacotherapy in patients enrolled in the study

Variable	All group (*n* = 25)	SR (*n* = 9)	AF (*n* = 16)
**Basic characteristics**			
Hypertension	20 (80)	7 (78)	13 (81)
Hypercholesterolemia	23 (92)	9 (100)	14 (88)
Type 2 diabetes	13 (52)	2 (22)	11 (69)
Obesity	7 (28)	1 (11)	6 (38)
Coronary artery disease	14 (56)	6 (67)	8 (50)
Atrial fibrillation	16 (64)	0 (0)	16 (100)
Chronic kidney disease	14 (56)	3 (33)	11 (69)
HF etiology			
Ischemic	13 (52)	6 (67)	7 (44)
Dilated	7 (28)	1 (11)	6 (38)
Idiopatic	5 (20)	2 (22)	3 (19)
NYHA class			
I	0	0	0
II	6 (24)	3 (33)	3 (19)
III	16 (64)	4 (44)	12 (75)
IV	3 (12)	2 (22)	1 (6)
**Laboratory evaluation**			
Creatinine, µmol/l	126 (49–329)	92.3 (67–122)	145.3 (49–329)
eGFR, ml/min/1.73 m2	58 (16–98)	73.1 (50–93)	49.1 (16–98)
Glucose, mmol/l	5.5 (3.8–7.8)	5.5 (4-6.9)	5.5 (3.8–7.8)
HbA1c, %	6.5 (5.4–10.1)	6.8 (5.4–10.1)	6.4 (5.5–9.3)
LDL‑C, mmol/la	2 (1-3.7)	2.2 (1.1–3.5)	1.8 (1-3.7)
HDL‑C, mmol/la	1.1 (0.6–1.8)	1.1 (0.6–1.4)	1.2 (0.7–1.8)
TC, mmol/la	3.4 (1.9–6.2)	3.7 (2.1–6.2)	3.3 (1.9-5)
Non‑HDL, mmol/la	2.3 (1.1–5.2)	2.6 (1.3–5.2)	2.1 (1.1-4)
TG, mmol/la	1.3 (0.6–5.5)	1.6 (0.8-5.5)	1.1 (0.6–1.9)
hs‑CRP, mg/l	6 (0.7–53.3)	3 (0.7-6)	7.7 (0.9–53.3)
NT‑proBNP, pg/ml	4010 (80-10494)	3190 (80-8110)	4471 (745-10494)
WBC, 103/µl	6.8 (3.8–10.8)	7 (4.2–10.8)	6.7 (3.8–9.1)
RBC, 106/µl	4.4 (3.5–5.9)	4.7 (4.3–5.9)	4.3 (3.5–5.4)
Hb, g/dl	13.2 (9.7–16.9)	13.9 (9.7–16.9)	12.8 (10.3–15.5)
HCT, %	39.5 (29.7–51.5)	41 (29.7–51.5)	38.7 (33.2–48.4)
MCV, fl.	87.8 (57–101)	83.6 (57-99.4)	90.2 (83–101)
MCH, pg	29.8 (22.5–35.4)	29.5 (22.5–35.4)	30 (25.4–34.6)
MCHC, g/dl	33.4 (30.7–36.1)	33.9 (32.7–35.6)	33.2 (30.7–36.1)
RDW, %	15.9 (12.4–26.6)	15.9 (12.4–26.6)	15.8 (13-21.1)
PLT, 103/µl	189 (73–353)	147.2 (73–310)	213.6 (142–353)
PDW, fl.	14.8 (11.1–19.5)	14.65 (11.1–18.6)	14.8 (11.6–19.5)
MPV, fl.	11.6 (10.2–13.3)	11.5 (10.2–13)	11.7 (10.2–13.3)
PCT, %	0.2 (0.1–0.4)	0.17 (0.1–0.33)	0.25 (0.16–0.38)
**Pharmacotherapy**			
ASA	6 (24)	6 (67)	0
Clopidogrel	6 (24)	3 (33)	3 (19)
Ticagrelor	0	0	0
Prasugrel	0	0	0
DAPT	3 (12)	3 (33)	0
TAT	0	0	0
DOAC	17 (68)	2 (22)	16 (100)
Statins	20 (80)	7 (78)	13 (81)
ACE/ARB/ARNI	19 (76)	5 (56)	14 (86)
MRA	18 (72)	7 (78)	11 (69)
SGLT2i	14 (56)	4 (44)	10 (63)
β‑Blocker	22 (88)	8 (89)	14 (88)

ACEI, angiotensin‑converting enzyme inhibitor; ARB, angiotensin receptor blocker; ARNI, angiotensin receptor/neprilysin inhibitor; ASA, acetylsalicylic acid; AF, atrial fibrillation; DAPT, dual antiplatelet therapy; DOAC, direct oral anticoagulant; eGFR, estimated glomerular filtration rate; HbA1c, glycated hemoglobin; HCT, hematocrit; HDL‑C, high‑density lipoprotein cholesterol; hs‑CRP, high‑sensitivity C‑reactive protein; LDL‑C, low‑density lipoprotein cholesterol; MCH, mean corpuscular hemoglobin; MCHC, mean corpuscular hemoglobin concentration; MCV, mean corpuscular volume; MPV, mean platelet volume; MR, mitral regurgitation; MRA, mineralocorticoid receptor antagonist; NT‑proBNP, N ‑terminal pro–B‑type natriuretic peptide; NYHA, New York Heart Association; PCT, plateletcrit; PDW, platelet distribution width; PLT, platelet count; RBC, red blood cell count; RDW, red cell distribution width; SGLT2i, sodium‑glucose cotransporter 2 inhibitor; SR, sinus rhythm; TAT, triple antithrombotic therapy; TC, total cholesterol; TG, triglycerides; WBC, white blood count

### Routine laboratory assessment

Routine laboratory assessment was performed during Visit 1. Laboratory investigations of blood fibrin clot properties, clot permeation and lysis time were performed during Visit 1 and 2.

### Sample collection and routine laboratory tests

Sample collection and routine laboratory assessment were previously published. Briefly, a fasting blood sample of 25 milliliters was extracted from the antecubital vein. The processing of samples occurred within 30-minutes following blood collection. Serum levels of total cholesterol (TC), low-density lipoprotein cholesterol (LDL-C), high-density lipoprotein cholesterol (HDL-C), triglycerides (TG), glucose, and glycated hemoglobin (HbA1c) were quantified using the biochemical analyzer Cobas 6000™ (Roche, Germany). High-sensitivity C-reactive protein (hsCRP) was measured through latex nephelometry (Dade Behring, Marburg, Germany). Creatinine levels were determined using a routine laboratory technique and eGFR was calculated based on the CKD-EPI formula. A complete blood count (CBC), encompassing red blood cell count, white blood cell count, hemoglobin, hematocrit, red blood cell distribution width, platelet distribution width, and platelet count, was conducted through standard laboratory assessment (Sysmex XT2000i, Sysmex, Japan).

### Laboratory investigations of blood fibrin clot properties, clot permeation and Lysis time

Thrombin generation, clot permeation, and CLT are used as representative coagulation parameters that provide a comprehensive, physiologically relevant picture of a patient’s thrombotic or fibrinolytic status that goes far beyond traditional clotting tests [21, 22, 23]. The assessment of these parameters was described previously. Briefly, venous blood was collected from the antecubital vein between 7:00 and 09:00 AM using citrated tubes (9:1 ratio of 0.106 M sodium citrate; Monovette, Sarstedt, Nümbrecht, Germany). The collected blood was subsequently subjected to centrifugation at 2500 g and 20 °C for 20 min, yielding platelet-poor plasma. This plasma was promptly snap-frozen within 30 min of collection and stored in aliquots at −80 °C until the time of analysis. For individuals receiving direct oral anticoagulants (DOACs) from whom blood was collected within the preceding 24 h, citrated plasma was treated with DOAC‑Stop (Haematex Research, Sydney, Australia) pri ‑ or to assessment [24].

### Fibrin permeation analysis

Fibrin clot permeation was determined using a pressure-driven system as described previously [24]. Briefly, 20 mM calcium chloride and 1 U/mL human thrombin (Merck KGaA, Darmstadt, Germany) were added to 120 µl citrated plasma. After 2 h of incubation in a wet chamber, tubes containing the clots were connected via plastic tubing to a reservoir of a buffer (0.01 M Tris, 0.1 M NaCl, pH 7.5) and its volume flowing through the gels was measured within 60 min. A permeation coefficient (Ks), which indicates the pore size, was calculated from the equation: Ks = QxLxη/txAxΔp, where Q is the flow rate in time t; L, the length of a fibrin gel; η, the viscosity of liquid; A, the cross-sectional area and Δp, a differential pressure.

### Plasma clot Lysis assay

The fibrinolysis capacity was assessed utilizing a clot lysis time (CLT) assay recommended by the International Society on Thrombosis and Haemostasis (ISTH) Subcommittee, as detailed previously [25]. In brief, citrated plasma was combined with 15 mM calcium chloride, 0.5 U/mL human thrombin (Merck), 15 µM phospholipid vesicles (Rossix, Mölndal, Sweden), and 18 ng/mL recombinant tissue plasminogen activator (rtPA, Boehringer Ingelheim, Germany). Subsequently, the mixture was transferred to a microtiter plate, and its turbidity was measured at 405 nm at a temperature of 37 °C. The CLT was defined as the duration from the midpoint of the clear-to-maximum-turbid transition, denoting clot formation, to the midpoint of the maximum-turbid-to-clear transition, signifying the lysis of the clot.

### Thrombin generation

Thrombin generation kinetics were assessed using the Calibrated Automated Thrombogram (CAT, Thrombinoscope BV, Maastricht, the Netherlands) following the manufacturer’s instructions. The measurements were conducted in a 96-well plate fluorometer (Ascent Reader, Thermolabsystems OY, Helsinki, Finland) equipped with the 390/460 filter set at a temperature of 37 °C. In summary, 80 µL of platelet-poor plasma were diluted with 20 µL of a reagent containing 5 pM recombinant tissue factor (TF), 4 micro-molar phosphatidylserine/phosphatidylcholine/phosphatidylethanolamine vesicles, and 20 µL of FluCa solution (HEPES, pH 7.35, 100 nM CaCl2, 60 mg/mL bovine albumin, and 2.5 mM Z-Gly-Gly-Arg-7-amino-4-methylcoumarin).

The Peak thrombin represents the maximum concentration of thrombin formed during the recording time, while the area under the curve corresponds to the endogenous thrombin potential (ETP). Lag time delineates the initiation phase of coagulation, and time to peak (ttPeak) signifies the propagation phase of thrombin generation. Each plasma sample underwent duplicate analysis, and the intra-assay variability was determined to be 8%.

### Statistical analysis

Continuous variables were presented as mean ± standard deviation (SD) or median (interquartile range, IQR), while categorical variables were expressed as numbers (%). The normality of distributions was assessed using Shapiro-Wilk tests. Inter-group differences for continuous variables were determined using Students t-test or Mann-Whitney U test. Pearson or Spearman rank correlation coefficients were calculated for associations between continuous variables with normal or non-normal distributions, respectively. Sample size was calculated for a 90% probability of detecting 15% differences in fibrin clot properties between groups at a p-value of 0.05, consistent with prior research [26–28]. To achieve that level of statistical power, each group should have at least 23 patients. Statistical analysis was performed using GraphPad Prism version 8.0.1 (San Diego, CA, USA) and IBM SPSS ver. 28.0 (Armonk, NY, USA).

## Results

### Group characteristics

Baseline characteristics of the study population is presented in Table 2. Patients who fully completed both visits were included in the final analysis (*n* = 25 pairs). Study population consisted of 16 males (64%) and 9 females (36%) with a mean age of 70.04 ± 8.01 years. All patients had previously diagnosed HF and severe MR eligible for TEER procedure. Secondary MR was identified in 80% of patients, while primary MR was observed in 20%. The majority of patients had pre-existing conditions such as hypertension (80%), hypercholesterolemia (92%), atrial fibrillation (AF; 64%) and type 2 diabetes (52%). The median observation time in this study was 50 days. Routine laboratory assessment and baseline study group characteristics are shown in Table 2.

Being aware that the presence of AF significantly affects both the assessment of LAS and coagulation system parameters, we divided the studied population into two groups: patients with sinus rhythm (SR) during the examination (*n* = 9) and those with AF (*n* = 16).

In a group with AF more patients in relative values had hypercholesterolemia (81% vs. 78%), type 2 diabetes (69% vs. 22%), obesity (38% vs. 11%) and chronic kidney disease (69% vs. 33%). All routine laboratory assessment and baseline characteristics are also depicted in Table 2.

### Echocardiography including left atrial strain analysis

All analyzed patients successfully underwent TEER. A single clip was implanted in 12 cases (48%), while two clips were used in the remaining ones. During follow up mitral regurgitation was graded as none/trace in 1 patients (4%), mild in 19 patients (79%) and moderate in 4 patients (17%).

Two post-procedural complications were observed. One patient developed a hematoma at the vascular access site, requiring intravenous antibiotic therapy. Another patient experienced self-limiting ventricular tachycardia (VT) right after the procedure. Due to recurrent VT episodes and HFrEF, this patient was subsequently considered for implantable cardioverter-defibrillator therapy.

In a whole group, during the 50-day follow-up period post-TEER, a significant reduction in LAS-cd was observed, while LAS-ct was notably increased. There was also a significant rise in MV velocity time integral (VTI), MV peak velocity and MV VTI to left ventricular outflow tract (LVOT) VTI ratio. No changes in LVOT VTI itself were noticed. Other analyzed basic echocardiographic parameters, including LA area, LAVI, LVEDV, LVESV and LVEF did not differ before vs. after the TEER procedure. Additionally, right ventricular (RV) size measured in PLAX significantly decreased. There were no notable changes in other parameters, including the size and function of the right atrium and ventricle. Complete description of echocardiography assessment is available in Table 3. We also confirmed a significant correlation between both baseline LAS-r and LAS-cd with MV VTI (Supplementary material, Figs. S1 and S2).

**Table 3:** Baseline echocardiographic evaluation in patients enrolled to the study (*n* = 25) and 6–8 weeks after TEER (*n* = 25)

Echocardiography parameters (*n* = 25)			
Variable	Baseline	6–8 weeks after TEER	*P* value
LVDD (PLAX), mm	65.1 (± 9)	65.7 (± 10.2)	0.69
LVEDV, mL	223 (± 67.7)	208.7 (± 68.7)	0.13
LVESV, ml	135 (107.5–203)	140 (83–190)	0.52
EF (BP Simpson), %	31 (28–40)	31.5 (21–46)	0.22
LA diameter, mm	57.1 (± 7.9)	56.1 (± 8.1)	0.41
LA area, cm2	39.8 (± 10.3)	39.4 (± 9.7)	0.78
LAVI, ml/m2	77.5 (71–102)	96 (69.5-110.5)	0.23
MR grade			
None/trace	0 (0)	1 (4)	
Mild,	0 (0)	19 (79)	
Moderate	0 (0)	4 (17)	
Severe	25 (100)	0 (0)	
MVA, cm2		3,09 (± 0.89)	
MV mean pressure gradient, mmHg	2 (1–2)	3 (2–5)	0.0002
MV VTI, cm	21 (18.8–25.9)	32.4 (25.8–38.9)	< 0.0001
LVOT VTI, cm	11.6 (± 2.5)	12.5 (± 2.8)	0.14
MV VTI/LVOT VTI ratio	2 (± 0.7)	2.7 (± 0.9)	0.00015
Peak MV velocity, m/s	1.2 (± 0.2)	1.4 (± 0.3)	0.0024
LAS-r, %	9.8 (6.6–12.3)	9.4 (7-12.5)	0.41
LAS-cd, %	−7.2 [(−9.4)-(−6.2)]	−5.4 [(−8.1)-(−3.8)]	0.03
LAS-ct, %	−2.2 [(−3.5)-(−0.8)]	−3.9 [(−6.2)-(−1.8)]	0.02
GLS-1, %	−9.2 [(−11.2)-(−7.4)]	−7.7 [(−11.4)-(−6.6)]	0.57
GLS-2, %	−10.4 [(−13.5)-(−7.4)]	−8.9 [(−12.2)-(−6.8)]	0.74
GLS-3, %	−9.3 [(−10)-(−7.3)]	−8.1 [(−10.8)-(−6.1)]	0.59
GLS-AVG, %	−9.5 [(−11.6)-(−7.5)]	−8.1 [(−12)-(−6.5)]	0.51
RVFWSL, %	−14.1 (± 3.6)	−16.7 (± 5)	0.45
RV4CSL, %	−11 (± 3.2)	−11.6 (± 4.7)	0.55
RV (PLAX), mm	35(31–42)	35 (32–38)	0.037
RV (Ap4Ch), mm	42 (37–46)	42 (40–47)	0.49
RA area, cm2	27.3 (± 8.6)	26.7 (± 7.9)	0.6
TAPSE, mm	19 (18–21)	20 (18–23)	0.77
TR V max, m/s	3 (2.5–3.3)	2.9 (2.7–3.1)	0.81
RVSP, mmHg	42 (33-49.5)	36 (32–43)	0.31
TAPSE/RVSP, mm/mmHg	0.44 (0.35–0.59)	0.5 (0.44–0.66)	0.96

EF, ejection fraction; GLS, global longitudinal strain; LAd, left atrial diameter; LAS-cd, left atrial strain in conduit phase; LAS-ct, left atrial strain in contraction phase; LAS-r, left atrial strain in the reservoir phase; LAVI, left atrial volume index; LVEDd, left ventricular end‑diastolic diameter; LVEDV, left ventricular end‑diastolic volume; LVESd, left ventricular end‑systolic diameter; LVEDV, left ventricular end‑diastolic volume; LVESV, left ventricular end‑systolic volume; LVOT VTI, left ventricular outflow tract velocity time integral; MR, mitral regurgitation; MV VTI, mitral valve tract velocity time integral; RA, right atrium; RV, right ventricle; RV4CLS, right ventricular four-chamber longitudinal strain; RVFWSL, right ventricular free wall longitudinal strain; RVSP, right ventricular systolic pressure; TAPSE, tricuspid annular plane systoli excursion; TR Vmax, tricuspid valve maximal velocity

Comparing two analyzed subgroups, we observed a significant difference in LA diameter and LA area, as well as in LAS-ct. There were no other significant differences between these two subgroups. All analyzed parameters and differences are presented in Supplementary material Table S1.

### Thrombin generation, clot permeation and Lysis time

In the whole cohort, all analyzed parameters of thrombin generation, KS and CLT did not show any significant differences and they are available in Table 4. During the analysis of the relationship between LAS and above mentioned blood fibrin clot characteristics, we found that baseline LAS-r showed a significant positive correlation with the change in CLT (*p* = 0.03; *R* = −0.43) before and after the TEER procedure (Fig. S2). We also analysed the correlations between the mitral valve area (MVA) assessed after the TEER procedure and coagulation parameters at each time point. Only one parameter, the endogenous thrombin potential (ETP) during follow-up, demonstrated a significant positive correlation with MVA (Table 5).

**Table 4:** Blood fibrin clot properties, clot permeation and Lysis time evaluation in patients enrolled to the study and 6–8 weeks after TEER (*n* = 25)

Coagulation parameters			
Variable	Baseline	6–8 weeks after TEER	*P* value
Ks 10^−9 cm2	4.27 (± 2.3)	5 (± 2)	0.12
Lag time, min	2.8 (2.1–3.7)	3 (2-3.7)	0.6
ETP, nM•min	1672 (± 378.2)	1650.4 (± 371.6)	0.83
Peak thrombin, nM	297.8 (± 85.2)	311 (± 103.1)	0.59
Time to peak, min	5.8 (± 1.7)	5.8 (± 1.9)	0.99
CLT, min	126 (103–158)	133 (112–160)	0.29
Fibrinogen, g/L	3.3 (± 0.7)	3.2 (± 0.6)	0.4

CLT, clot lysis time; ETP, endogenous thrombin potential; Ks, permeation coefficient; Lag time, time to initiate thrombin generation; Peak, peak thrombin generation; Time to peak, time to peak thrombin generation

**Table 5:** Correlations analysis between MVA measured after TEER and coagulation parameters

	MVA after TEER	
Baseline		
Ks	R = 0.216	p = 0.36
Lagtime	r = −0.173	p = 0.47
ETP	R = 0.278	p = 0.235
Peak	R = 0.374	p = 0.105
Time to Peak	R = −0.206	p = 0.383
CLT	r = −0.308	p = 0.186
Fibrynogen	R = −0.242	p = 0.334
**6–8 weeks after TEER**		
Ks	R = 0.5374	p = 0.82
Lagtime	R = 0.02809	p = 0.91
**ETP**	**R = 0.4537**	p = **0.044**
Peak	R = 0.1525	p = 0.52
Time to Peak	R = 0.1577	p = 0.51
CLT	r = 0.055	p = 0.818
Fibrynogen	R = −0.1645	p = 0.488
**Change in parameters (∆)**		
Ks	R = 0.1969	p = 0.4
Lagtime	r = −0.3713	p = 0.11
ETP	r = 0.07218	p = 0.76
Peak	r = 0.1624	p = 0.49
Time to Peak	R = −0.3418	p = 0.14
CLT	R = −0.3225	p = 0.1655
Fibrynogen	r = −0.2095	p = 0.404

Data shown as R, Pearson coefficient correlation; r, Spearman coefficient correlation; p, p-value for correlation analysis

CLT, clot lysis time; ETP, endogenous thrombin potential; Ks, permeation coefficient; Lag time, time to initiate thrombin generation; MVA, mitral valve area; Peak, peak thrombin generation; Time to peak, time to peak thrombin generation

Distinct differences were observed between patients with SR and AF in several blood fibrin clot properties, such as clot permeation characteristics (Ks), lag time, and time to peak. Additionally, a significant difference was noticed in fibrinogen level post procedure. There were no changes in examined blood fibrin clot characteristics before and after the procedure. Detailed results are provided in Supplementary Material Table S2.

**Fig. 2:**
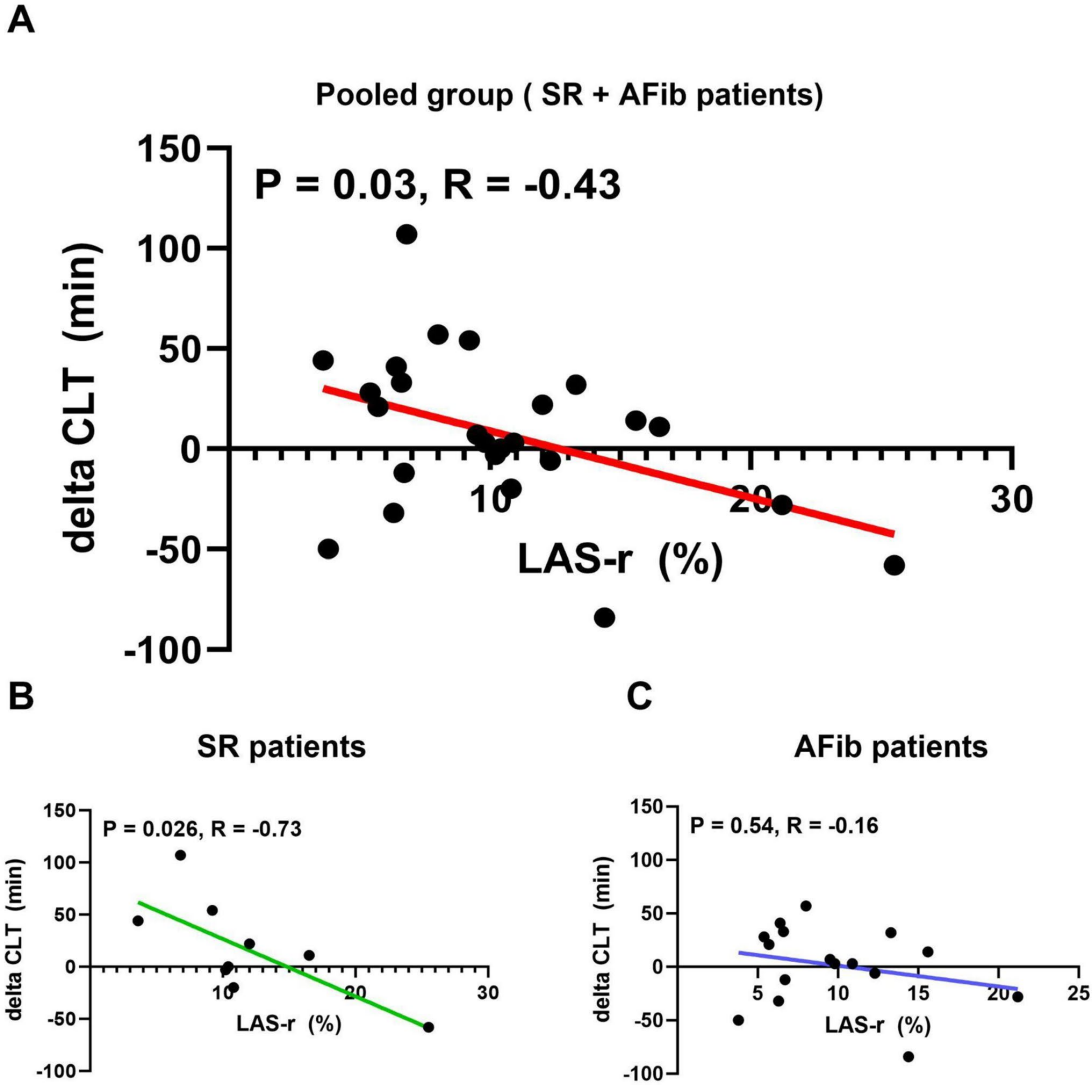
Associations between baseline LAS-r and change of CLT: **(A)** after 6–8 weeks observation following TEER; **(B)** in patients with SR; **(C)** in patients with AF. Data are demonstrated as linear regression with Mean and Error. Abbreviations: AFib - atrial fibrillation; CLT - clot lysis time; LAS-r - reservoir phase of the left atrium; SR - sinus rhythm

We conducted a similar analysis of the relationship between LAS and fibrin clot characteristics in patients with SR and AF. In patients with SR, a significant association was found between LAS-r and CLT (*p* = 0.73; *R* = 0.026). However, no statistically significant association was observed in patients with AF. This result is illustrated in Fig. 2. Furthermore, patients with SR showed a correlation with LAS-cd, while those with AF exhibited an association with LAS-ct. The correlations between each LAS parameter and changes in CLT for these patients are presented in Table 6.

**Table 6:** Correlation analysis of LAS-r with change in CLT in patients with sinus rhythm (SR) and atrial fibrillation (AF)

	SR	AF
	∆CLT	∆CLT
LAS-r	R = 0.73 p = 0.026	*R* = 0.25 *p* = 0.35
LAS-cd	R = −0.84 p = 0.0049	*R* = 0.07 *p* = 0.79
LAS-ct	R = −0.26 p = 0.5	r = −0.56 p = 0.025

Data shown as R, Pearson coefficient correlation; r, Spearman coefficient correlation; p, p-value for correlation analysis

LAS-cd, left atrial strain in conduit phase; LAS-ct, left atrial strain in contraction phase; LAS-r, left atrial strain in the reservoir phase; R - Pearson correlation coefficient, r - Spearman correlation coefficient

## Discussion

In this investigation, our focus was directed towards evaluating a novel approach for assessing LA function, involving LAS in conjunction with thrombin generation, clot permeation and lysis time in patients diagnosed with HF and severe MR undergoing TEER. Our main findings demonstrate a positive correlation between alterations in CLT subsequent to mitral TEER and the level of baseline LAS-r. We also observed a marked reduction in LAS-cd and a notable increase in LAS-ct during the 50-day follow-up period, as well as a decrease in RV size.

TEER induces transient activation of the coagulation cascade, evidenced by short-term elevations in prothrombin fragment 1 + 2 and thrombin–antithrombin III complexes, without platelet activation [29]. It was shown that these markers normalized within a month, especially after anticoagulation initiation [29]. Contrary, dual antiplatelet therapy does not reduce thrombotic risk and increases bleeding, whereas short-term anticoagulation more effectively suppresses coagulation activation [29].

Some meta-analyses and registry data support at least 4 weeks of anticoagulation post-TEER to reduce stroke and thromboembolism risk without increasing major bleeding [30]. However, adding antiplatelet agents raises bleeding risk without additional benefit [30]. Therefore, the optimal post-TEER antithrombotic strategy remains undefined.

### Left atrial strain and thromboembolic risk

Until now, no published study has established a direct correlation between LAS and fibrin clot properties, clot permeation or lysis time. Although the association between LAS and prothrombotic risk has been extensively documented in numerous studies. For instance, Mannina et al., in their cohort study, demonstrated that decreased positive longitudinal LAS (PALS, which is equivalent to the LAS-r) and increased negative longitudinal LAS rate (LASR, which refers to the rate at which the distance between two points in the myocardium changes throughout the cardiac cycle) were independently linked with incidence of the ischemic stroke in elderly adults without previously diagnosed AF or cerebrovascular events [31, 32]. A study by Vera et al. observed that lower levels of LAS-r were associated with an increased rate of stroke recurrence or mortality due to cryptogenic stroke [16]. Another investigation performed in the setting of an acute HF and SR, demonstrated that reduced LAS-r was correlated with enhanced susceptibility to stroke (annual stroke incidence rate of 2.38%) [17].

We also observed a significant increase in MV VTI, peak MV velocity, and the MV/LVOT VTI ratio in patients following TEER. This ratio was previously described by Isabel G. Scalia in her research, where she reported that patients with an elevated MV/LVOT VTI ratio exhibited higher rates of all-cause mortality, cardiac-specific mortality, and heart failure-related hospitalizations at one year post-TEER. These findings suggest that the MV/LVOT VTI ratio may serve as a valuable prognostic indicator for identifying patients at increased risk of adverse outcomes following TEER, emphasizing the importance of enhanced surveillance and individualized management strategies in the post-procedural period [33].

The morphology and function of the left atrium (LA) are crucial factors in determining thrombotic risk. Reduced LA function can result in blood stasis, one of the components of Virchow’s triad [34, 35]. Additionally, a dilated and stretched LA may contribute to atrial wall fibrosis, which is linked to endothelial dysfunction—another element of Virchow’s triad—further increasing the risk of thrombus formation [34]. All above mentioned mechanisms could contribute to unfavorable changes in fibrin clot properties.

Given the anticipated differences in blood fibrin clot properties, clot permeation and LAS between patients with and without AF, we stratified the cohort into two subgroups to determine whether these differences could influence the study endpoints. The analysis revealed a significant difference in LA size and LA area, likely attributable to remodeling and fibrosis associated with AF [36]. Furthermore, the AF subgroup exhibited significantly lower LAS-ct values. Ashikhmin et al. demonstrated in his research that patients with paroxysmal AF, even while in sinus rhythm (SR), exhibit markedly reduced atrial contractile function [37].

Regarding coagulation parameters, patients in SR had significantly higher Ks, suggesting a larger pore size between fibrin fibers, which may contribute to a lower prothrombotic risk [38]. These patients also exhibited a shorter lag time to fibrin clot formation and a reduced time to reach peak fibrin concentration. However, the interpretation of these two parameters remains uncertain, as they have been shown to vary considerably in previous studies.

Additionally, we analyzed the two subgroups concerning the primary outcome of this study—the association between baseline LAS-r and changes in CLT. Notably, this correlation was significant only in patients with SR, who also showed a positive correlation with LAS-cd. In contrast, patients with AF demonstrated a positive correlation with LAS-ct, as those patients have less evident or not present contractile function of LA [39].

The extensive documentation of the correlation among fibrin clot permeability, clot lysis, and thrombin generation includes studies not only in patients with HF [13, 40]. Exploring the relationship between baseline LAS-r and changes in CLT after TEER could significantly contribute to further research involving larger populations. Understanding how these factors interact is crucial for clarifying the mechanisms of thrombotic disorders and developing targeted therapies.

### Left atrial strain in heart failure

Moreover, other studies demonstrate the correlation between LAS and HF. In a systematic review and meta-analysis of 7787 patients irrespective of ejection fraction levels, asserts that PALS emerged as a predictor for both all-cause mortality and cardiac hospitalization in HF patients [41]. This underscores the clinical significance of LAS in estimating the prognosis of HF population. Additionally, LAS showed its prognostic significance in patients with acute HF, where lower baseline LAS and lack of its improvement after optimal treatment was associated with higher risk of rehospitalization and cardio-vascular events. These findings were also shown by Bouwmeester et al. in patients with HF without AF or severe renal failure [42].

Moreover, Aga et al. demonstrated that in patients with HF with reduced ejection fraction (HFrEF), where echocardiographic estimation of left atrial pressure was not feasible due to the unavailability of a reliable E/A ratio, the assessment of LAS-r may offer incremental clinical and prognostic utility, particularly in the context of monitoring disease progression or exacerbation [43].

### Left atrial strain and mitral regurgitation

LAS shows its importance also in research regarding patients with MR. Recent research demonstrated that impaired PALS was linked to diminished LV function and increased severity of functional MR [44].Moreover, in individuals with HFrEF and functional MR, PALS independently predicted all-cause mortality and provided further information beyond conventional LA and LV measurements [44]. Additionally, it was found that functional alterations in the LA function precede its dilation, potentially serving as an early indicator of LA remodeling [45]. This characteristic could be utilized as an early prognostic marker in patients with MR, distinguishing those who require more intensive treatment or monitoring [45].

Finally, LAS-r and LAS-ct appear to play a more significant role in the cardiac cycle, particularly in patients with decreased LV relaxation [10]. T. Sugimoto et al. sought to determine which phase of LAS was most influenced by MR. It was found that LAS-r and LAS-ct were crucial in the abnormal physiological response to exercise [11]. Consequently, MR repair by e.g.: TEER procedure, might help enhance the contractile function of the LA. However, Romano et al. observed that patients with AF after TEER showed improvement in LAS-cd, which contrasts with the findings of our study [46]. This may be attributed to the significantly different follow-up periods, during which left atrial remodeling occurred.

Alterations in RV dimensions and functionality subsequent to TEER have been documented in prior investigations. Neuser et al. conducted a study examining the impact of TEER on the right heart chambers, noting a potential association between less impaired RV strain and increased likelihood of its recovery following MR treatment [47]. Similar to our findings, the authors did not identify that tricuspid annular plane systolic excursion (TAPSE) was a reliable predictor of right ventricular function following TEER. Notably, TAPSE has exhibited diverse outcomes across various studies, thereby rendering its reliability questionable in predicting RV dysfunction within this patient cohort [47–50].

Future research should conduct a larger, multicenter study to validate the link between LAS—especially LAS-r—and fibrin clot properties in TEER patients with severe MR. The goal is to confirm LAS-r as a predictive biomarker for prothrombotic risk, find meaningful threshold values, and assess the prognostic significance of LAS in major outcomes like stroke and cardiovascular mortality. Additionally, the study should evaluate if LAS-guided stratification improves outcomes over standard management and examine the safety of anticoagulation strategies tailored to LAS and clot profiles, aiming to reduce thromboembolic events without more bleeding. Comprehensive imaging, including transesophageal echocardiography and left atrial appendage flow assessment, is crucial for accurate thrombotic risk characterization and patient selection refinement.

## Limitations

While the findings of this study align with existing research, it is important to acknowledge its limitations. The small sample size, particularly the limited number of patients in sinus rhythm, reduces statistical power and generalizability, and limits the ability to control for confounding variables such as age, comorbidities, and medication use. Additionally, the inclusion of patients with diverse heart failure phenotypes and both functional and structural mitral regurgitation introduces heterogeneity, which may affect the consistency of the findings across subgroups. Moreover, we did not perform transesophageal echocardiography assessments of left atrial appendage flow, spontaneous echo contrast, or pulmonary vein flow before or after the procedure. These measurements could have provided important information on localized thrombotic risk, as prior research suggests that prothrombotic changes may be more pronounced in the left atrial appendage in structural heart disease. Lastly, the absence of long-term follow-up limits the ability to link the observed changes in atrial strain and clot properties with clinical thromboembolic events.

## Conclusions

Patients with heart failure and severe mitral regurgitation undergoing TEER, changes in LAS, particularly reservoir strain (LAS-r), are significantly associated with alterations in fibrin CLT, suggesting a potential interplay between cardiac mechanical function and prothrombotic status. This association is especially evident in patients with sinus rhythm, where impaired LAS-r correlates with prolonged CLT—a marker of hypofibrinolysis. These findings highlight LAS as a promising tool for thrombotic risk assessment and underscore the importance of left atrial function monitoring post-TEER.

## Supplementary Information

Supplementary Material 1

## Data Availability

Upon justified request, we are able to provide access to our scientific database.

## Abbreviations

AF: Atrial fibrillation
CLT: Clot lysis time
ETP: Endogenous thrombin potential
Ks: Permeation coefficient
LAS: Left atrial strain
LAS-cd: LAS conduit
LAS-ct: LAS contractile
LAS-r: LAS resevoir
LVOT: Left ventricle output tract
MV: Mitral valve
MVA: Mitral valve area
MR: Mitral regurgitation
SR: Sinus rythm
TEER: Transcatheter edge-to-edge repair
VTI: Velocity time integral
