# Supplementary material for: Association of left atrial strain and fibrin clot properties in patients with heart failure and severe mitral regurgitation undergoing transcatheter edge-to-edge repair
Source: J Thromb Thrombolysis. 2025 Aug 29;59(2):272–85. doi: 10.1007/s11239-025-03169-0 (PMC13017979; doi:10.1007/s11239-025-03169-0)
Supplement: Supplementary file 1 — Supplementary Material 1 [file 11239_2025_3169_MOESM1_ESM.docx]

**Supplementary Table 1.** Baseline echocardiographic evaluation in patients with sinus rhythm (SR) and atrial fibrillation (AF).

| **Echocardiography parameters** | | | |
| --- | --- | --- | --- |
| **Variable** | **SR** | **AF** | **p** |
| LVDD (PLAX), mm | 66.4 (± 7.7) | 65.3 (± 10.1) | 0.763 |
| LVEDV, mL | 240.3 (± 76.9) | 225.7 (± 80) | 0.668 |
| LVESV, ml | 167.7 (± 62.5) | 146.2 (± 60.8) | 0.465 |
| EF (BP Simpson), % | 30 (26-48.5) | 31.5 (26.5-42.3) | 0.77 |
| **LA diameter, mm** | **54 (51.5-57.0)** | **61 (54.0-63.8)** | **0.04** |
| **LA area, cm2** | **34.3 (± 4.3)** | **42.5 (± 11.3)** | **0.049** |
| MR volume, mL | 49 (± 15.3) | 53.2 (± 14.9) | 0.511 |
| LAVI, ml/m2 | 74.5 (± 14.1) | 95.1 (± 31.8) | 0.1 |
| MV VTI, cm | 23 (±4.8) | 21.7 (±7.6) | 0.52 |
| LVOT VTI, cm | 11 (±3) | 11.9 (±2.2) | 0.38 |
| MV VTI/LVOT VTI | 2.3 (1.7-2.6) | 1.8 (1.4-2) | 0.12 |
| Peak MV velocity, m/s | 1.2 (±0.2) | 1.2 (±0.2) | 0.56 |
| LAS-r, % | 11.7 (± 6.3) | 9.7 (± 4.6) | 0.39 |
| LAS-cd, % | -8.2 (±4.7 ) | -7.5 (± 3) | 0.663 |
| **LAS-ct, %** | **-3.47 (2.46)** | **-1.73 (1.47)** | **0.04** |
| GLS-1, % | -7.6 (-15.1-[-6,13]) | -10.2 (-11.2-[-8.7]) | 0.14 |
| GLS-2, % | -9.9 (± 4.9) | -11.2 (± 4) | 0.51 |
| GLS-3, % | -7.55 (-13.9-[-6.85]) | -9.6 (-10.0-[-8.3]) | 0.26 |
| GLS-AVG, % | -7.45 (-14.1-[-7.13]) | -10.2 (-11.7-[-8.75]) | 0.14 |
| RVFWSL, % | -12.9 (± 4.2) | -14.7 (± 3.2) | 0.264 |
| RV4CSL, % | -9.8 (± 3.2) | -11.4 (± 2.9) | 0.231 |
| RV (PLAX), mm | 35.2 (± 7.5) | 36 (± 7.9) | 0.812 |
| RV (Ap4Ch), mm | 41.2 (± 7.1) | 43 (± 7.6) | 0.573 |
| RA area, cm2 | 24 (± 5.6) | 28,6 (± 9.4) | 0.192 |
| TAPSE, mm | 19 (17.5-25) | 18.5 (18-21) | 0.59 |
| TR V max, m/s | 2.8 (2.7-3.2) | 3.1 (2.5-3.4) | 0.62 |
| RVSP, mmHg | 39.3 (10.7) | 44.6 (15.7) | 0.4 |
| TAPSE/RVSP, mm/mmHg | 0.0784 (0.0592-0.0844) | 0.0673 (0.061-0.0833) | 0.67 |

**Data shown as mean (**±**SD) or median (IQR)**

**Caption to Supplementary Material, Table 1.** Abbreviations: EF, ejection fraction; GLS, global longitudinal strain; LAd, left atrial diameter; LAS-cd, left atrial strain in conduit phase; LAS-ct, left atrial strain in contraction phase; LAS-r, left atrial strain in the reservoir phase; LAVI, left atrial volume index; LVEDd, left ventricular end‑diastolic diameter; LVEDV, left ventricular end‑diastolic volume; LVESd, left ventricular end‑systolic diameter; LVEDV, left ventricular end‑diastolic volume; LVESV, left ventricular end‑systolic volume; LVOT VTI, left ventricular outflow tract velocity time integral; MR, mitral regurgitation; MV VTI, mitral valve tract velocity time integral; RA, right atrium; RV, right ventricle; RV4CLS, right ventricular four-chamber longitudinal strain; RVFWSL, right ventricular free wall longitudinal strain; RVSP, right ventricular systolic pressure; TAPSE, tricuspid annular plane systoli excursion; TR Vmax, tricuspid valve maximal velocity.

**Supplementary Table 2.** Baseline fibrin clot properties, clot permeation and lysis time in patients in patients with sinus rhythm (SR) and atrial fibrillation (AF).

| **Coagulation parameters** | | | |
| --- | --- | --- | --- |
| **Pre-procedure** | | | |
| **Variable** | **SR (n=9)** | **AF (n=16)** | **p** |
| **Ks 10^-9 cm2** | **5.8 (± 2.4)** | **3.4 (± 1.8)** | **0.011** |
| **Lagtime, min** | **2.56 (1.67-2.95)** | **3.17 (2.48-4.25)** | **0.03** |
| ETP, nM•min | 1688.5 (± 421.6) | 1662.8 (± 365.8) | 0.874 |
| Peak thrombin, nM | 324.6 (± 76.2) | 282.7 (± 88.5) | 0.245 |
| **Time to peak, min** | **4.9 (± 1.3)** | **6.3 (± 1.8)** | **0.046** |
| CLT, min | 104 (94.5-188.0) | 127 (104-156) | 0.84 |
| Fibrinogen, g/L | 3 (± 0.6) | 3.4 (± 0.7) | 0.154 |
| **Post-procedure** | | | |
| **Variable** | **SR (n=9)** | **AF (n=16)** | **p** |
| **Ks 10^-9 cm2** | **6.19 (±2.26)** | **4.29 (±1.44)** | **0.016** |
| **Lagtime, min** | **2.35** **(±0.68)** | **3.47 (±1.28** | **0.025** |
| ETP, nM•min | 1648.63 (1500.58-1826.76) | 1776.66 (1711.55-2057.33) | 0.19 |
| Peak thrombin, nM | 349.61 (±71.43) | 289.34 (±113.54) | 0.17 |
| Time to peak, min | 4.95 (±1.34) | 6.28 (±1.99) | 0.09 |
| CLT, min | 130 (115-157) | 135.5 (107-161) | 0.89 |
| **Fibrinogen, g/L** | **2.76 (±0.55)** | **3.44 (±0.52)** | **0.006** |
| **Change post/pre-procedure (delta)** | | | |
| Ks 10^-9 cm2 | 0.47 [(-0.54)-0.79] | 0.8 (0.47-1.44) | 0.27 |
| Lag time, min | -0.01 (±0.5) | 0.007 (±1.78) | 0.97 |
| ETP, nM•min | -27.3 (-251.86)-166.72 | -3.3 [(-103.99)-53.05] | 0.63 |
| Peak thrombin, nM | 13.79 [(-11.29)-59.54] | -2.08 [(-110.625)-63.41] | 0.42 |
| Time to peak, min | 0.06 (±0.82) | -0.04 (±2.6) | 0.91 |
| CLT, min | 17.4 (±47.36) | 1.7 (±36.54) | 0.36 |
| Fibrinogen, g/L | -0.17 (±0.25) | -0.07 (±0.7) | 0.68 |

**Data shown as mean (**±**SD) or median (IQR)**

**Caption to Supplementary material, Table 2. Abbreviations:** CLT, clot lysis time; ETP, endogenous thrombin potential; Ks, permeation coefficient; Lag time, time to initiate thrombin generation; Peak, peak thrombin generation; Time to peak, time to peak thrombin generation.

**Supplementary Figure 1.** Associations between baseline LAS-r and MV VTI: **A.** In the whole cohort; **B.** in patients with SR; **C.** in patients with AF.


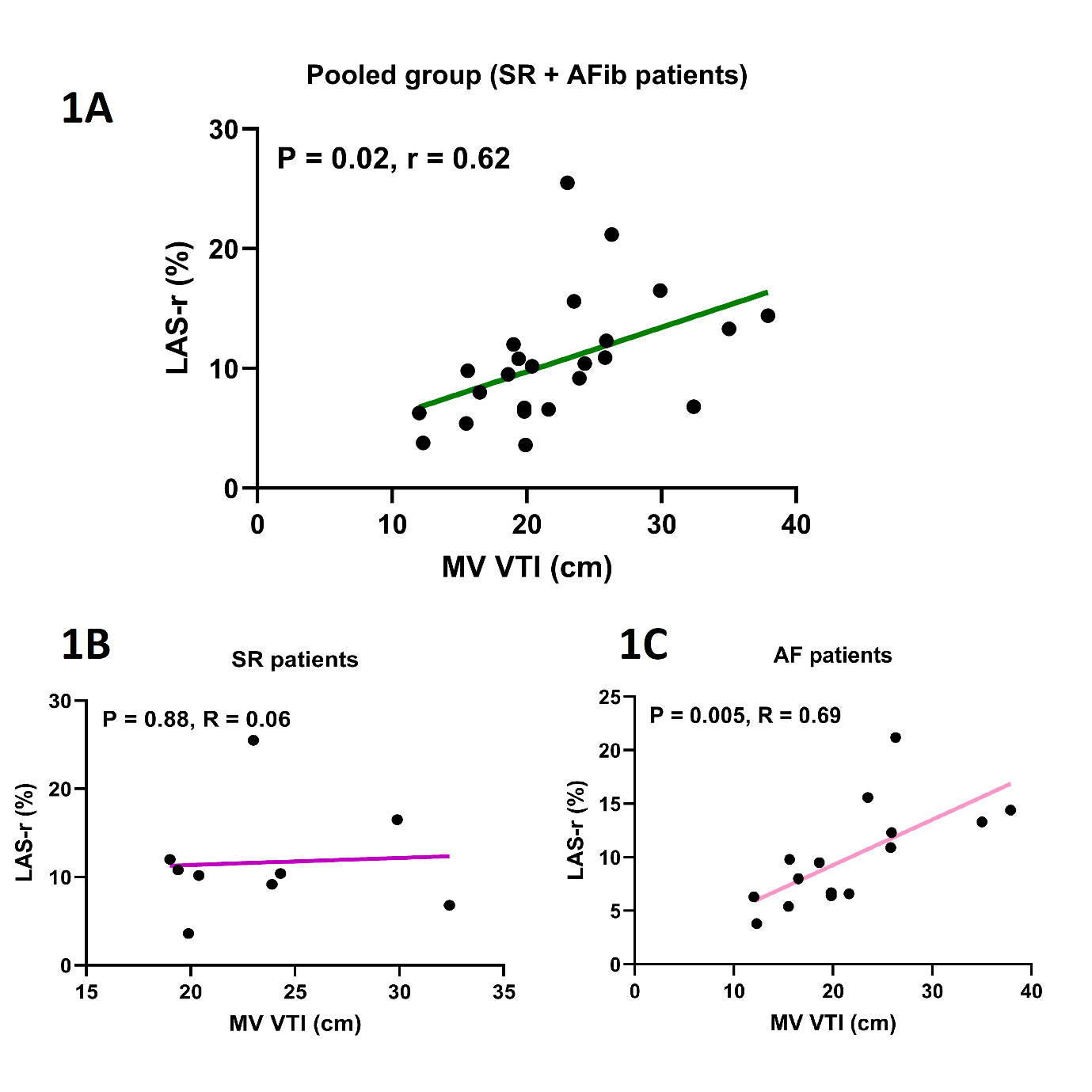


**Captions to Supplementary Figure 1**. Data are demonstrated as linear regression with Mean and Error. Abbreviations: AFib - atrial fibrillation; LAS-r - reservoir phase of the left atrium; MV VTI – mitral valve velocity time integral; SR - sinus rhythm

**Supplementary Figure 2.** Associations between baseline LAS-cd and MV VTI: **A.** In the whole cohort; **B.** in patients with SR; **C.** in patients with AF.


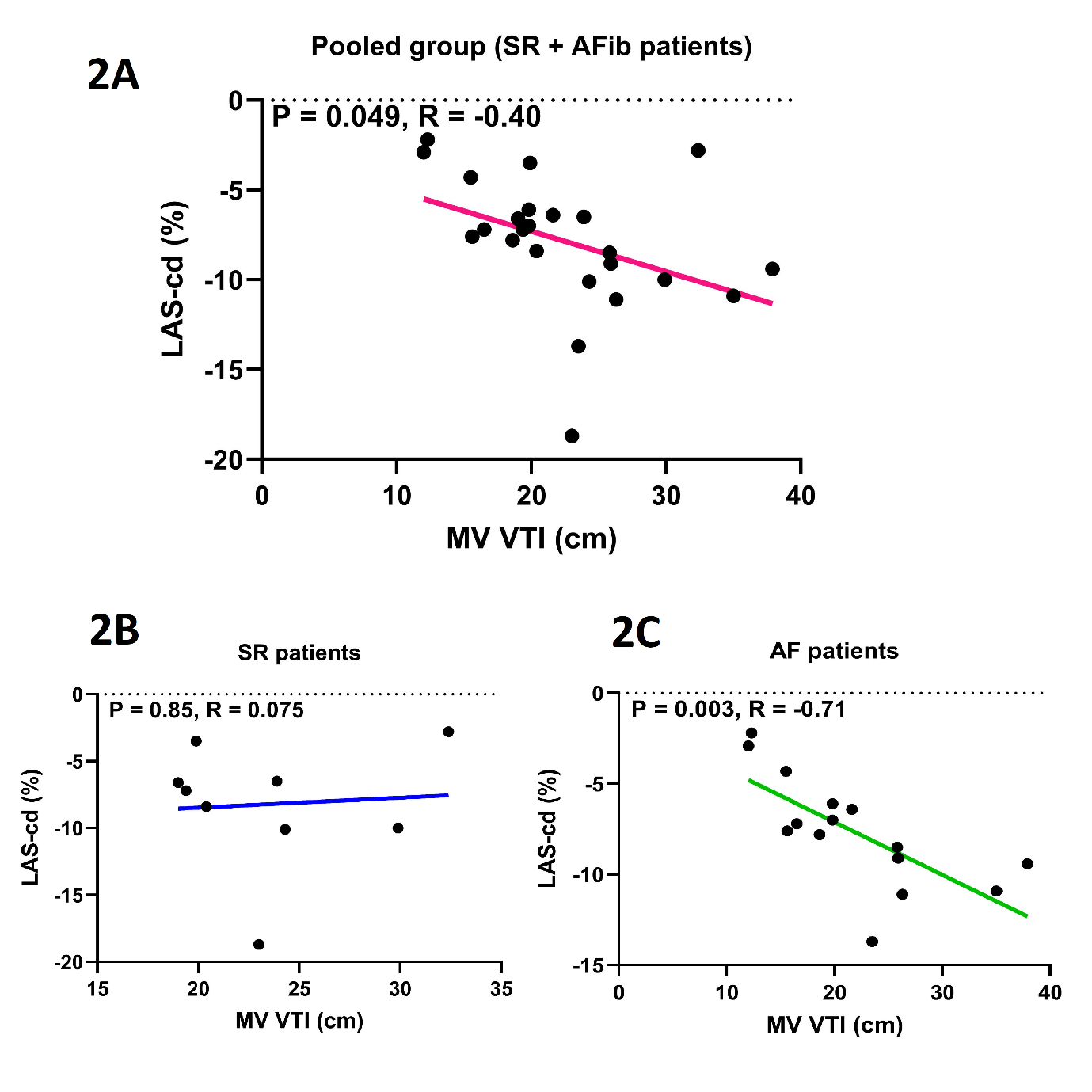


**Captions to Supplementary Figure 2**. Data are demonstrated as linear regression with Mean and Error. Abbreviations: AFib - atrial fibrillation; LAS-cd – conduit phase of the left atrium; MV VTI – mitral valve velocity time integral; SR - sinus rhythm
